# The Role of the Patient Information Leaflet in Patients'Medication Therapy: A Case Study within the Kumasi Metropolis of Ghana

**DOI:** 10.1155/2020/2489137

**Published:** 2020-10-15

**Authors:** Frederick W. A. Owusu, Genevieve Naana Yeboah, Rahel Adutwiwah Aboagye, Cedric Dzidzor K. Amengor, Philomena Entsie

**Affiliations:** ^1^Department of Pharmaceutics, School of Pharmacy, Central University, Accra, Ghana; ^2^Department of Pharmaceutical Sciences, Faculty of Health Sciences, Kumasi Technical University, Kumasi, Ghana; ^3^Centre for Plant Medicine Research, Akuapem-Mampong, Ghana; ^4^Department of Pharmaceutical Sciences, Royal Ann College of Health, Kumasi, Ghana; ^5^Department of Pharmaceutical Chemistry, School of Pharmacy, University of Health and Allied Sciences, Ho, Ghana; ^6^Department of Herbal Medicine, Faculty of Pharmacy and Pharmaceutical Sciences, Kwame Nkrumah University of Science and Technology, Kumasi, Ghana

## Abstract

One of the tools used in providing comprehensible medication information to patients on their medication use for improved adherence and subsequent optimal therapeutic effect is the Patient Information (PI) leaflet. In Ghana, the patient information leaflet is available through various sources including health-care professionals (HCPs) and electronic forms. The World Health Organization (WHO) estimates that more than 70% of patients, especially in the developing countries, who receive medications do not read the accompanying leaflet. This study assessed the role of the patient information leaflet in Patients' medication therapy in the Kumasi metropolis of Ghana. A random cross-sectional survey was conducted in various hospitals and pharmacies within selected districts in the Kumasi metropolis. The survey revealed that 96.9% of the sampled respondents (*n* = 300) were provided with PI leaflets on their medicines while only 3.1% of them indicated otherwise. Among the proportion of respondents who were provided with PI leaflets, 66.7% of them read the information on the drug leaflets whilst the remaining 33.3% did not. Ultimately, 62.4% of those who read the PI leaflets were influenced to discontinue their medication. In conclusion, reading of the drug information leaflet was higher than that found in previous studies in Ghana. Reading the leaflet did not increase adherence but aroused anxiety and decreased adherence in some patients. A large number of the patients who were given the PI leaflets indicated that it did not provide them with the needed information.

## 1. Introduction

The need for high-quality written information for patients about their medications is well established in literature [1, 2]. The Patient Information (PI) leaflet is a piece of standardized written information about the safe and effective use of a prescription or specified over-the-counter medicine prepared by pharmaceutical manufacturers in one or more of the three formats: package inserts, loose leaflets, and electronic. Many countries require Patient Information (PI) leaflets about drug therapy to be included in the medication package, and the content of these is mandated by regulatory guidelines, with Ghana being no exception [3]. The issue of PI leaflets has drawn many criticisms over the years, from consumer representative groups, health-care professionals (HCPs), pharmaceutical manufacturers, and Government bodies. Based on a patient-centered approach, the aim of a written patient information leaflet is to support patients in tasks such as decision making and/or taking medication correctly [4]. More than ever, consumers now want to know about their medicines and their impact in order to make informed choices. Providing patients with informative, well-set out leaflets which are easy to navigate can lead to improved quality of life, reduced anxiety, early recognition of adverse side effects, and clearer understanding of the treatment regimen. The World Health Organization defines medication adherence as the degree to which a person's behavior corresponds with the agreed recommendations from a health-care provider [5, 6]. One major factor that influences adherence is the patient's ability to read and understand medication instructions and its accompanying leaflet. Adherence to treatments is a key determinant of effective diagnosis. Failure to conform is a major issue that affects not only the patient but also the health-care system. Nonadherence to medicine in patients leads to severe deterioration of illness, mortality, and increased cost of healthcare. Despite PI leaflets being available in Ghana for over 15 years, research undertaken in 2004 suggested that less than 30% of consumers received a PI leaflet. The formation of committees such as the Patient Engagement Advisory Committee (PEAC), along with the development and availability of usability guidelines and core PI leaflet templates for PI leaflet writers, has improved and standardized the patient information leaflet document [7, 8]. However, these have not resulted in a significant increase in utilization and provision by HCPs or consumers. The introduction of remuneration for pharmacists, repeated reports, and exhortations by pharmacy bodies has had little impact on increasing PI leaflet provision. Consumers are still largely unaware of their existence despite consumer health representative bodies lobbying heavily for action to increase consumer awareness. Written medicine information in conjunction with verbal counselling has proven to have a positive impact on consumers and may aid in increasing knowledge, satisfaction, and adherence to medicine therapy [9]. This study, therefore, sought to assess the role of the PI leaflet in Patients' medication therapy in the Kumasi metropolis of Ghana and to provide current information on the prevalence of the PI leaflet use amongst patients.

## 2. Materials and Methods

### 2.1. Study Area

A random cross-sectional survey was conducted in various hospitals and pharmacies within four selected districts in the Kumasi metropolis.

### 2.2. Period of Study

The study was conducted between the period of November 2018 and May 2019.

### 2.3. Questionnaire Pretesting

A pilot survey was carried out at Atwima Manhyia in the Atwima Nwabiagya Municipality. To avoid bias, random respondents (males and females; literates and illiterates) were selected within the municipality. Fifty (50) questionnaires were issued to these respondents and were read out to those who cannot read nor write. The consent of participants was sought prior to administration of questionnaires. The duration, competency, and suitability of the questionnaires (time frame for answering of questions, ease of understanding the questions and answers, appropriateness of questions to survey objectives) were pretested, and these produced Cronbach's alpha of 0.916, an indication that the questionnaires were well structured.

### 2.4. Sample Size Selection

The sample size was determined based on the population size for the four districts selected [10]. A total of 300 participants were involved.

### 2.5. Sampling

Participants were randomly selected to avoid bias and included both men and women who were, at least, sixteen years at the time of sampling. The consent of participants was sought prior to administration of questionnaires.

### 2.6. Data Collection Method

The questionnaires were given to both male and female and were read out in local Akan dialect to patients who could not read nor write. Questionnaires were retrieved the same day from participants to avoid questionnaire loses. Questionnaires were collated and analyzed using Statistical Package for the Social Science (SPSS).

## 3. Results

### 3.1. Sociodemographics of Respondents

We investigated the sociodemographics of the respondents to determine their ages and sex. A majority of the sampled respondents (39%) were between the ages of 21 to 30, giving a fair idea of the age demographics within the metropolis who patronized health-care facilities. Notably, 52% of these respondents where females while males were 48%. Almost all the age groups had majority of respondents in the group being female with the exception of the respondents belonging to the 31–40 years group (Table 1).

### 3.2. Respondents' Sources of Information on the Prescribed Medicines

Patients' information on their prescribed medication can be obtained from varied sources. Taking this into account, the sampled respondents were grouped according to four information sources: pharmacy staff, doctor, friend, and relative. The study uncovered that majority (37.6%) of the participants obtained information about medicines from pharmacy staff, 32.3% from friends, and 23.6% from relatives, with doctors being the least source of information with a frequency of 6.3% (Table 2).

### 3.3. Provision and Purpose of the PI Leaflet

**Figure 1:**
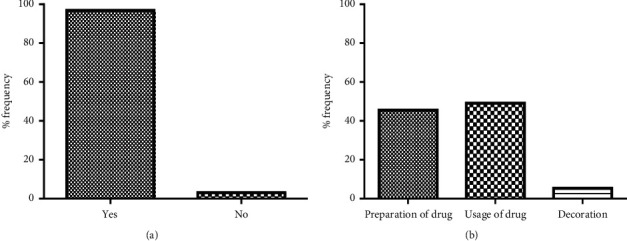
(a) Provision of the drug leaflet on medication to respondents. (b) Perception of respondents about the purpose of PI leaflets.

The study also sought information on respondents being given drug leaflets on their current/last medication, as well as their understanding of the purpose of such leaflets (Figures 1(a) and 1(b)). The results (Figure 1(a)) show that 96.9% of the sampled respondents were provided with drug leaflets on the medicines they bought while only 3.1% of them indicated otherwise. A majority (49.1%) of the respondents indicated that PI leaflets provide information on how their medicines will be taken, while only few (5.4%) indicated that the PI leaflet was for a decorative effect (Figure 1(b)).

### 3.4. Reading of the Drug Leaflet and Its Effects on Respondents' Adherence to Medication

The survey further revealed that majority (66.7%) of the respondents who were provided with the PI leaflets read the information on the drug leaflets whilst 33.3 % of them indicated otherwise.

The participants who read the PI leaflets were asked if there is any instance, where they will stop taking their medication because of some information from the leaflet. A majority (62.4%) responded yes with 37.6% of them responding no (Table 3).

To show two components pertaining to respondents recommending that every patient be given a PI leaflet and that this leaflet be written in their local dialect, a majority of the respondents (78.4%) who read the PI leaflet indicated that they will recommend PI leaflets to be given to other patients to enhance their decision on whether to take their medications or not. A minority of the respondents (21.6%) indicated otherwise (Table 4). In terms of recommending information in the local dialect, the results showed that a greater proportion of the respondents (80.4%) recommend that the information on the drug leaflet be written in the local dialect, as compared to 19.6% of them who did not.

## 4. Discussion

This current study was to investigate the role of the patient information leaflet in patients' medication therapy. The patient information leaflet serves as a useful document that informs and guides medicine users and/or their caretakers on their medication. It was observed from the study that majority (52%) of the sampled respondents were females. This was because the females availed themselves to provide the needed responses compared to the males who indicated that they did not have time to answer the questionnaires. Furthermore, females are known to frequent health-care facilities more often than their male counterparts [11].

Sources of medicine information include medical professionals, Internet, and relatives among others. The quality of the received information may vary from source to source. It is, therefore, very important to encourage patients to obtain information on their medicines from the right sources. This study revealed that despite individuals having multiple sources of obtaining information about a particular drug, the most dominant source of information was the pharmacy staff. This might be due to the fact that pharmacy staff including pharmacists are well trained medical professionals who have adequate knowledge about drugs prescribed to individuals and, hence, can provide the requisite information for patients [12]. In the hospitals, pharmacists are usually the last health-care professionals to interact with the patient before discharge and, hence, are required to provide the patient with all the needed information on their prescribed drugs. Again, at the community pharmacy, it is expected that the pharmacist remains the sole source of interaction with the patient and the primary source of information on the prescribed drugs [13, 14]. Thus, the pharmacist being the lead pharmacy staff has an unparalleled role to play in ensuring that PI leaflets are appreciated and well utilized by patients. This will reduce the proportion of the populace who rely on friends and relatives for information concerning their medications. Patients may not obtain the accurate and needed information from friends and relatives, and this will have a negative impact on patients' adherence to their medication therapy [15].

There is an increase in the percentage of patients who are provided with PI leaflets as compared to previous studies where only 30% of patients who visited health-care facilities in Ghana were provided with a PI leaflet [3]. The rapid increase in the provision of PI leaflets to patients can be attributed to strict enforcement of PI leaflets in drug packaging by the FDA, requisition of PI leaflets by patients due to increase in awareness by health-care practitioners and regulatory agencies and a general increase in the literacy rate among the general populace. Again, advertisement and increase in awareness of the presence and significance of PI leaflets by the FDA and health-care professional are responsible for this observed percentage distribution [16]. Although majority of respondents know that the PI leaflet provides information on how to use the medications, a few think it is for decorative purposes. There is, therefore, the need for regulatory bodies such as the FDA to continue to educate the general population about the function and importance of these leaflets.

Increase in the literacy rate among the general populace is a major reason why a majority of the populace read the PI leaflet. According to the recent census (2010) conducted by the Ghana Statistical Services, the literacy rate for the population 15 years and older stands at 71.5% with 45.1% being literate in both the English and Ghanaian language. The Ashanti region recorded 15.7% literacy rate for the population 15 years and older with 11.0% being literate in both the English and Ghanaian language [10]. However, illiteracy (5%), lack of time (2%), bulky nature (16.3%), and complexity and unattractiveness (10%) of the PI leaflet were the majors reasons given by respondents for not reading the PI leaflet. An important factor to consider in designing of the leaflets is its readability, as well as the ease of understanding. To ensure this, easily comprehendable legible text should be used in the creation of the patient information leaflet [17].

It was evident that majority of the respondents were likely to stop taking a particular medicine due to the information on the drug leaflet. Reasons provided by the respondents for not taking their medications were the information provided on the leaflet showed that the drug had more side effects compared to its benefits (15%), some of the information on the PI leaflet were scary (40%), and the complex nature of the information on the PI leaflet (7.4%). This might be as a result of the difficulty encountered in reading or understanding the language in which the information was written. According to [17], in 15% out of 219 specialists who expressed their opinions in the free text field, the frequently criticized problems on patient information leaflets were lack of comprehensibility and clarity. Nearly, one-third of these specialists were of the view that package inserts unsettles patients and subsequently lead to noncompliance. Thus, the FDA and other stakeholders involved in regulating the PI leaflet need to enhance the PI template and interface for patient convenience. More guidelines on good patient information leaflet design to help make these leaflets patient centred must be put in place [18]. Again, increase in font size of the content on patient information leaflets can have a positive effect on clarity and legibility of information. This will reduce nonadherence of medication therapy by patients due to the PI leaflet.

Furthermore, majority of the respondents who received patient information leaflets recommended that it should be provided to all patients. A majority of respondents also indicated that the information on drug leaflets should be written in the local dialect to ensure ease of understanding. This further corroborates the importance of PI leaflets in drug therapy.

## 5. Conclusions

The study revealed that majority (96.9%) of the respondents were given drug leaflets, and the proportion of patients reading the patient information leaflet is 66.7%, which is higher than in previous studies. The study also showed that majority of respondents who received PI leaflets recommend that these leaflets be made available to patients. Attention should be drawn to that fact that having the PI leaflets written in the local dialect to enhance patient's understanding is desired and recommended.

## Figures and Tables

**Table 1:** Sociodemographic details of respondents.

		Sex of respondents		Total
		Male	Female	
Age group of respondents	16–20 years	22 (44.9%)	27 (55.1%)	49 (16.3%)
	21–30 years	58 (49.6%)	59 (50.4%)	117 (39.0%)
	31–40 years	50 (58.1%)	36 (41.9%)	86 (28.7%)
	40 years and above	14 (29.2%)	34 (70.8%)	48 (16.0%)
Total		114 (48.0%)	156 (52.0%)	300 (100%)
Inability to read and write		7 (2.33%)	10 (3.33)	17 (5.66%)

**Table 2:** Respondents' sources of information about medication.

Response	Frequency	Percentage (%)
Pharmacy staff	113	37.6
Doctor	19	6.3
Friend	97	32.3
Relative	71	23.6
Total	300	100

**Table 3:** Influence of leaflet information on respondents' decision to stop taking the medication.

Response	Frequency	Percentage (%)
Yes	121	62.4
No	73	37.6
Total	194	100

**Table 4:** Respondents' recommendations on patients being given similar information leaflets and leaflets being written in the local dialect.

		Respondents' recommendation of the leaflet being written in the local dialect		Total
		Yes	No	
Respondents' recommendation on patients being given similar leaflets	Yes	141 (72.6%)	11 (5.8%)	152 (78.4%)
	No	15 (7.8%)	27 (13.8%)	42 (21.6%)
Total		156 (80.4 %)	38 (19.6 %)	194 (100%)

## Data Availability

The data used to support the findings of this study are available from the corresponding author upon request.

## References

[1] Dickinson D., Raynor D. K. (2003). Ask the patients—they may want to know more than you think. *BMJ*.

[2] Grime J., Blenkinsopp A., Raynor D. K., Pollock K., Knapp P. (2007). The role and value of written information for patients about individual medicines: a systematic review. *Health Expectations*.

[3] Ankrah D. N. A., Ofei C. N. (2010). The effect of advice to read the medicine/patient information leaflet among patients in Ghana: a cross-sectional study. *Journal of Pharmaceutical Health Services Research*.

[4] Koo M., Krass I., Aslani P. (2006). Enhancing patient education about medicines: factors influencing reading and seeking of written medicine information. *Health Expectations*.

[5] Dickinson D., Raynor D. K., Duman M. (2001). Patient information leaflets for medicines: using consumer testing to determine the most effective design. *Patient Education and Counseling*.

[6] Julius R. J., Novitsky M. A., Dubin W. R. (2009). Medication adherence: a review of the literature and implications for clinical practice. *Journal of Psychiatric Practice*.

[7] Food and Drugs Authority *Guidelines for Registration of Allopathic Drugs*.

[8] Food and Drugs Authority *Guidelines for Patient’s Information Leaflets*.

[9] Ane M. G. (2018). Ghana strives for a more humane drug policy. *GDPO Situation Analysis*.

[10] Ghana Statistical Service (2010). *Population and Housing Census Report*.

[11] Redondo-Sendino A., Guallar-Castillon P., Banegas J. R., Rodriguez-Artalejo F. (2006). Gender differences in the utilization of health-care services among the older adult population of Spain. *BMC Public Health*.

[12] Hughes L., Whittlesea C., Luscombe D. (2002). Patients’ knowledge and perceptions of the side-effects of OTC medication. *Journal of Clinical Pharmacy and Therapeutics*.

[13] Oshikoya K. A., Oreagba I., Adeyemi O. (2011). Sources of drug information and their influence on the prescribing behaviour of doctors in a teaching hospital in Ibadan, Nigeria. *Pan African Medical Journal*.

[14] World Health Organization (2002). *Promoting Rational Use of Medicines: Core Components (No. WHO/EDM/2002.3)*.

[15] Askehave I., Zethsen K. K. (2014). A comparative analysis of the lay-friendliness of Danish EU patient information leaflets from 2000 to 2012. *Communication and Medicine*.

[16] Kyei S., Ocanse S., Koffuo G. A., Abokyi S., Feni K. A. (2014). Patients’ information leaflets: its’ influence on ophthalmic patient education and medication compliance. *British Journal of Medicine and Medical Research*.

[17] Fuchs J., Banow S., Gorbert N., Hippius M. (2007). Importance of package insert information in the European union. *Die Pharmazeutische Industrie*.

[18] Young A., Tordoff J., Smith A. (2018). Regulatory agencies’ recommendations for medicine information leaflets: are they in line with research findings?. *Research in Social and Administrative Pharmacy*.

